# Ga-68-PSMA PET/CT ? or PET/MRI ?, Mp-MRI ? in diagnosis and radiotherapy planning in a patient with prostate cancer

**DOI:** 10.1590/S1677-5538.IBJU.2020.0519

**Published:** 2020-09-02

**Authors:** Yasemin Benderli Cihan

**Affiliations:** 1 Department of Radiation Oncology Kayseri City Hospital Turkey Department of Radiation Oncology, Kayseri City Hospital, Turkey

To the editor,

Prostate cancer (PC) has a highly variable clinic. It can remain for an extended period of time without any findings, as well as shows an aggressive course. Early diagnosis is very important. The most important diagnostic methods used in the PC are digital rectal examination, transrectal ultrasonography and prostate specific antigen (PSA) values. The exact diagnosis is made by histopathology ( 1 ). The correct staging of the PC is very important as it directly affects the treatment decision and patient management. Currently, staging tests are not recommended since the risk of metastasis is low in patients with low risk compared to D’Amico risk classification. It is recommended that patients in the middle-high risk group be performed by abdominal computer tomography (CT) or magnetic resonance imaging (MRI) with bone scintigraphy for staging ( 2 - 4 ).

Since the 1980s, MRI has been used in the evaluation of the prostate gland and its surrounding structures among radiological diagnostic methods. It was originally used for staging in patients with PC diagnosis, and for determining invasion and lymph node metastases. Conventional MRI examinations (especially T2-weighted examinations) are the basic method for detecting PC, but they have high sensitivity but low specificity ( 2 ). In recent years, new software and techniques in MRI technique have made progress in anatomical, functional and physiological evaluation. Thus, the evaluation has increased sensitivity and specificity. In addition to high-resolution T2A examinations, dynamic, diffusion and MRI spectroscopies have been added to the diagnosis of PC. The MRI technique performed by adding at least two functional MRIs to the T2A sequences is called Multiparametric MRI (Mp-MRI). This method is the most commonly used technique for prostate imaging today. It is especially recommended to use MRI device with 3 Tesla main magnet power in imaging. Compared to 1.5T tesla devices, the signal-to-noise ratio, temporal and spatial resolution are higher in 3T devices. Biopsies can be taken from the lesion described in the light of Mp-MRI, and this reduces false negative rates ( 4 - 6 ). One of the most important benefits of Mp-MRI is its extraprostatic extension and local recurrence. Because the extension outside the capsule and the seminal vesicle involvement counted in the extraprostatic extension criterion are independent pathological criteria that increase the risk of local recurrence, progression and death. The probability of local recurrence in these cases considered high risk is 40-50% ( 6 , 7 ). According to the meta-analysis of 5681 cases by de Rooij et al; in extracapsular invasion, MRI sensitivity and specificity were found to be 57% and 91%, respectively ( 2 ). In a study by Pokorny et al. they compared transrectal ultrasound guided biopsy and MP-MRI guided biopsy. They found that the MP-MRI examination reduced the biopsy requirement by 51%. They also reported that the MP-MRI examination reduced the clinical significance of low prostate cancer by 89.4% and increased the detection of medium / high risk prostate cancer by 17.7%. ( 3 ). In a review, it is stated that Mp-MRI is found to be highly specific and highly sensitive in detecting local recurrences and in the diagnosis of bone and nodal metastasis, even in patients with low PSA levels (0.3-0.5 ng / mL) ( 5 ).

Ga-68 PSMA (Prostate Specific Membrane Antigen) PET / CT and PET / MRI examination, which has been on the agenda in recent years, shows promising results and is predicted to be effective in improving the management of patients with prostate cancer ( 1 , 6 , 7 ). PET / MRI works on the basis of developing fusion of PET with Mp-MRI for diagnostic purposes in oncological applications. PET / MRI reporting requires higher experience than PET-CT. Because these devices are expensive, they are used in a limited number of academic centers. Instead, fusion Ga 68 PSMA PET and abdominal MRI images made at different times are thought to be guiding. MRI evaluates soft tissue involvement and bone lesions, while PET provides biological information about cancer; it is superior in distinguishing the residual-recurrent tumor ( 6 - 8 ). In the study, Mp-MRI is found superior in terms of resolution to CT and PET / CT in the “T” staging of primary prostate malignancies ( 7 ). Kitajima et al. report that Mp-MRI is superior in their study involving 115 patients and compared 11C-choline PET/CT and Mp-MRI imaging in detecting recurrent prostate cancer after radical prostatectomy ( 8 ).

The treatment options created according to the risk groups in PC treatment according to D’Amico risk classification include hormonal therapy, external radiotherapy (RT), brachytherapy and radical prostatectomy. In recent years, the use of RT has increased significantly in PC therapy. Non-invasive imaging methods used in multiparametric MRI, 68 Gallium PSMA PET / CT and PSMA PET / MRI in the diagnosis and post-treatment follow-up of PC have been preferred in recent years ( 1 , 5 , 6 , 9 ). In treatment planning, it is not clear which imaging method to prefer and its superiority from each other. In a study comparing PSMA-PET / CT and Mp-MRI in determining gross tumor volume in patients who planned to have radiotherapy after radical prostatectomy, Betterman and colleagues found that the median tumor volume was smaller than Mp-MRI (p <0.05). Sensitivity and specificity were 86% and 87% for PSMA-PET, 58% and 94% for Mp-MRI. As a result, it was reported that determining RT tumor volume is more important than PSMA-PET/CT ( 6 ). Jambor and colleagues compared 18F-FACBC PET/CT, PET/MRI and Mp-MRI to their potential to detect intraprostatic disease and pelvic lymph nodes in terms of detection and characterization of PC in patients undergoing radical prostatectomy. It was not seen that all three tests had superiority in diagnosis. Also, PET / MRI and Mp-MRI were unable to detect pelvic lymph node metastases less than 8 mm. They stated that 18F-FACBC PET / MRI should be preferred in primary tumor detection and focal ablative therapeutic approaches are planned ( 4 ). In another study, the reliability of 68Ga-PSMA-PET/ CT, CT and PET / CT prostate cancer was investigated which of the tests performed to make radiotherapy decision. They reported that 68Ga-PSMA-PET/CT is more sensitive in lesion detection compared with conventional CT ( 9 ).

As a result, prostate cancer is a cancer that can be extremely heterogeneous. It can remain silent for a long time, or behave very aggressively. Therefore, it is important to be able to determine the tumor behavior as well as the diagnosis. Because excessive increases in diagnosis cause diagnosis and excessive treatment of tumors that are clinically insignificant, which may remain silent for many years, these can be prevented by distinguishing clinically insignificant cancers and cancers that may progress aggressively. Today, in the diagnosis, it has been found that the use of PSA and derivatives used in conjunction with digital rectal examination reduces prostate cancer-specific mortality, while the number of biopsies has increased by 70-80%. This has led to the need for advanced imaging methods, reducing the number of biopsies and leading to targeted biopsies. One of the advanced imaging methods, 68 Ga PSMA PET / CT, PET / MRI or Mp-MRI has an important place in diagnosis, staging and treatment planning in prostate cancer. Currently, it is not known which imaging technique is superior and that it will be preferred more. Studies investigating the place of these techniques in diagnosis and treatment are needed.
